# Successful closure of a delayed persistent duodenal perforation using endoscopic suturing after unsuccessful surgical intervention

**DOI:** 10.1055/a-2474-7005

**Published:** 2024-11-29

**Authors:** Jameel Alp, Rahul Karna, Natalie Wilson, Mohammad Bilal

**Affiliations:** 1 5635 Department of Medicine, University of Minnesota, Minneapolis, United States; 2 5635 Department of Medicine, Division of Gastroenterology, Hepatology, and Nutrition, University of Minnesota, Minneapolis, United States; 3 20040 Gastroenterology & Hepatology, Minneapolis VA Medical Center, Minneapolis, United States

Various tools are available for endoscopic management of acute gastrointestinal tract perforations. However, managing delayed duodenal perforations endoscopically is challenging because of the risk of retroperitoneal contamination, duodenal angulation, fibrotic nature of ulcers, and the endoscope’s paradoxical motion 1 2 . This video presents a case of persistent duodenal perforation despite surgical intervention, which was ultimately managed successfully with endoscopy.

An 80-year-old man with a history of metastatic melanoma being treated with nivolumab, and immune checkpoint inhibitor-induced colitis, on steroid taper, presented with abdominal pain. A computed tomography (CT) scan of the abdomen revealed pneumoperitoneum and perforation at the anterior gastroduodenal wall. Urgent laparoscopy showed purulent peritonitis with omentum adhering to the stomach, suggesting the perforation site had sealed, and a Jackson–Pratt (JP) drain was placed. However, the patient continued to experience severe abdominal pain and bilious output in the JP drain. A subsequent CT scan with water-soluble oral contrast revealed a persistent duodenal leak and increased pneumoperitoneum. After multidisciplinary discussion, the patient was referred to the advanced endoscopy team ( Video 1 ).

Endoscopic closure of duodenal perforation by use of a full-thickness suturing device.Video 1

Esophagogastroduodenoscopy performed on postoperative day 8 revealed a large duodenal ulcer (15 mm) on the right lateral wall at the junction of the first and second duodenal portions, with a focal defect consistent with perforation. Argon plasma coagulation was used to ablate the ulcer edges to induce granulation. Endoscopic closure was achieved using a full-thickness suturing device (OverStitch Endoscopic Suturing System; Apollo, Austin, Texas, USA) in a “Z” configuration, and the ulcer bed was closed. A small area at the edge was closed with through-the-scope endoclips. A nasoduodenal tube was placed and connected to low intermittent wall suction. Follow-up imaging showed no contrast extravasation, confirming resolution of the leak, and the patient transitioned to enteral nutrition and was discharged. The JP drain was removed after 2 weeks.

Endoscopy_UCTN_Code_TTT_1AO_2AI

## Notes

Endoscopy E-Videos https://eref.thieme.de/e-videos
*E-Videos* is an open access online section of the journal *Endoscopy* , reporting on interesting cases and new techniques in gastroenterological endoscopy. All papers include a high-quality video and are published with a Creative Commons CC-BY license. Endoscopy E-Videos qualify for HINARI discounts and waivers and eligibility is automatically checked during the submission process. We grant 100% waivers to articles whose corresponding authors are based in Group A countries and 50% waivers to those who are based in Group B countries as classified by Research4Life (see: https://www.research4life.org/access/eligibility/ ). This section has its own submission website at https://mc.manuscriptcentral.com/e-videos .

## References

[1] Lee JH Kedia P Stavropoulos SN AGA Clinical practice update on endoscopic management of perforations in gastrointestinal tract: Expert review Clin Gastroenterol Hepatol 2021 19 2252 226100 10.1016/j.cgh.2021.06.045 34224876

[2] Ye L Wang Y Hou W Endoscopic partial closure followed by adequate drainage for treating delayed perforation caused by duodenal endoscopic submucosal dissection: A case report Medicine (Baltimore) 2019 98 e15883 10.1097/MD.0000000000015883 31145346 PMC6708997

